# Association of the Systemic Immune-Inflammatory Index, Neutrophil-to-Lymphocyte Ratio, and Platelet-to-Lymphocyte Ratio in the Differentiation of Chronic Otitis Media: A Prospective Observational Analytical Study

**DOI:** 10.7759/cureus.96526

**Published:** 2025-11-10

**Authors:** Shaila Sidam, Anjan K Sahoo, Aparna G Chavan, Vivek K Bharti

**Affiliations:** 1 Otolaryngology - Head and Neck Surgery, All India Institute of Medical Sciences, Bhopal, Bhopal, IND; 2 Otolaryngology, All India Institute of Medical Sciences, Bhopal, Bhopal, IND

**Keywords:** cholesteatoma, chronic otitis media, inflammation, mucosal, squamosal

## Abstract

Introduction: The systemic immune-inflammatory index (SII) is a biomarker derived from neutrophil, lymphocyte, and platelet counts obtained from a complete blood count. It has shown diagnostic and prognostic significance in various diseases. In chronic otitis media (COM), differentiation is essential preoperatively to determine the appropriate treatment. Hence, the purpose of this study was to assess the predictive value of inflammatory markers in differentiating mucosal and squamosal disease.

Objective: To evaluate the association of SII, neutrophil-to-lymphocyte ratio (NLR), and platelet-to-lymphocyte ratio (PLR) in the differentiation of chronic otitis media.

Methods: This was a prospective observational analytical study conducted from July 16, 2024, to July 17, 2025, in 100 cases of chronic otitis media. A detailed history was taken, and ear examination, hearing evaluation, and blood sample collection were performed to assess the complete blood count. The numbers of neutrophils, lymphocytes, and platelets were recorded, and NLR, PLR, and SII were then calculated in the two groups.

Results: Neutrophil and lymphocyte counts were higher in group one compared to group two, whereas the platelet count was higher in group two compared to group one. NLR was elevated in group two compared to group one. NLR, PLR, and SII were all elevated in group two compared to group one, with a statistically significant p-value of <0.001.

Conclusion: SII holds significant potential in differentiating between mucosal and squamosal types of chronic otitis media. As a simple, cost-effective, and easily obtainable parameter, SII demonstrated superior diagnostic performance compared to NLR and PLR.

## Introduction

Chronic otitis media (COM) is defined as mucosal inflammation of the middle ear and temporal bone lasting more than three months. It is classified into safe/tubotympanic/mucosal type and unsafe/atticoantral/squamosal type. Furthermore, it can be classified as active, which is discharging at the time of examination, and inactive, with no discharge for three to six months. It can cause hearing loss and perforation of the tympanic membrane. When there is an accumulation of keratinizing squamous epithelium in epithelial-lined air spaces of the middle ear and mastoid, it is called COM with cholesteatoma. Based on clinical presentation, it can be active COM with or without cholesteatoma or inactive COM [1]. Although many factors have been identified, such as downregulation of proinflammatory cytokines, neutrophil apoptosis, and activation of macrophages in studies conducted at the molecular level, the cause of tissue damage and the pathophysiology are still not fully understood [2,3]. The treatment for COM with cholesteatoma is tympanoplasty with mastoidectomy, and for inactive COM it is tympanoplasty [1]. Markers measuring inflammation are expensive, limiting their availability. Hence, laboratory examination is a cost-effective and routinely available option.

The systemic immune-inflammation index (SII) is a new biomarker derived from lymphocyte, neutrophil, and platelet counts in a complete blood count. It has shown diagnostic and prognostic efficacy in many diseases [4]. SII has emerged as a significant biomarker in evaluating systemic inflammation across a variety of conditions, particularly in malignancies, cardiovascular diseases, and infectious diseases [5]. Elevated SII levels have been linked to poorer prognoses in numerous cancer types, indicating their role as a potential prognostic tool [6]. In the context of infections, SII has been shown to correlate with disease severity and mortality in various conditions, including COVID-19, sepsis, and pneumonia, further highlighting its potential as a marker of inflammatory activity [7-9]. Its consistency across studies suggests that, while not perfect, SII can contribute meaningful insights into the assessment of inflammatory states and help guide clinical decision-making [5-12].

The neutrophil-lymphocyte ratio (NLR) is defined as the absolute number of neutrophils divided by the absolute number of lymphocytes, and the platelet-lymphocyte ratio (PLR) as the absolute number of platelets divided by the absolute number of lymphocytes [4]. Neutrophilia and lymphocytopenia, key immune system responses to systemic inflammation, injury, and stress, reflect a complex interplay of factors such as infection, trauma, major surgery, sepsis, and endocrine stress responses [13,14]. Neutrophils, crucial in the early phases of infection and immune regulation, are elevated during these conditions due to processes such as demargination, suppressed apoptosis, and the action of growth factors like G-CSF [14]. Lymphocytopenia, marked by reduced circulating lymphocytes, occurs in various stressful conditions involving tissue injury, apoptosis, and cytokine modulation. The simultaneous changes in neutrophil and lymphocyte counts during systemic inflammation highlight a multifactorial response involving immunologic, neuroendocrine, and biological factors [15,16]. This dynamic can be quantified using NLR, identified as an effective and cost-efficient severity parameter. NLR has gained prominence as a prognostic tool for assessing the severity of various critical conditions, including infections, malignancies, cardiovascular diseases, and respiratory failure [17-22]. An NLR value greater than 3 to 5 is often associated with poor clinical outcomes, and studies have demonstrated its utility in predicting mortality, cancer prognosis, and cardiac events [15].

Thrombocytosis and lymphocytopenia are both closely tied to systemic inflammation, with conditions such as sepsis, malignancy, trauma, and rheumatologic disorders triggering platelet proliferation through proinflammatory cytokines such as IL-6 and IL-1 [23]. Elevated platelet counts contribute to the exacerbation of inflammation by increasing blood vessel permeability and platelet activation [23]. Despite its potential, the PLR’s diagnostic utility remains limited due to challenges in determining optimal cutoff values, variations in diagnostic accuracy across ethnicities, and the retrospective nature of many studies.

Systemic inflammation can be measured using biochemical and hematological markers; however, these may be time-consuming as well as expensive. Measuring NLR and PLR is inexpensive, readily available, and reproducible. NLR and PLR can therefore be used as markers of chronic infection [4].

## Materials and methods

Study design and setting

This was a prospective diagnostic and analytical study conducted in the Department of ENT, Head and Neck Surgery, at a tertiary care institute in Central India from July 16, 2024, to July 17, 2025. Ethical approval was obtained from the Institutional Ethical Review Board before the commencement of the study (IHEC-LOP/2024/P24/048).

Study population

Patients presenting to the ENT outpatient department (OPD) with the chief complaint of chronic ear discharge for a duration of six weeks or more (COM) and aged above 18 years were enrolled in the study (Table 1). Written informed consent was obtained from all recruited study participants.

**Table 1:** Inclusion and exclusion criteria COM: chronic otitis media.

Type	Criteria
Inclusion	All COM cases above 18 years of age.
Exclusion	Patients with a history of acute otitis media cases, traumatic cases, previously operated cases, malignancy cases, those who had chronic inflammations, such as chronic heart disease, chronic lung disease, chronic kidney disease, autoimmune diseases, rheumatologic diseases, and neuromuscular diseases, and those who had psychological diseases were excluded from the study.

Diagnostic criteria of different types of COM are presented in Table 2 [24].

**Table 2:** Diagnostic criteria of COM The presence of otoscopic findings with or without other radiologic or radiologic features was taken as diagnostic criteria for differentiation of types COM. COM: chronic otitis media.

Criteria	Mucosal COM	Squamous COM (Cholesteatomatous)
Otoscopy	Central perforation, smooth edges, non-foul-smelling discharge	Attic perforation, irregular edges, foul-smelling discharge, keratin debris
Radiology	Mild mastoid opacification, no ossicular damage	Mastoid opacification, ossicular erosion, cholesteatoma cysts
Intraoperative	Inflamed but intact mucosa, preserved ossicles, no cholesteatoma	Cholesteatoma (keratin debris), ossicular erosion, granulation tissue, bony destruction

Sample size

Since the prevalence of COM in India is around 2% to 5% [25,26], the sample size was calculated using the following formula:

$n=\frac{Z^{2}\times p(1-p)}{e^{2}}$

where n is the sample size, Z is the Z-score corresponding to the desired confidence level (Z = 1.96 for 95% confidence level), p is the estimated proportion of the population (2%-5%), and e is the margin of error (5% in our study). Calculations revealed a sample size range of 31 to 73. Power was calculated using G*Power version 3.1.9.7 (Heinrich-Heine Universität Düsseldorf, Düsseldorf, Germany) for an effect size of 0.5 with the above-mentioned parameters, and the calculated power was 95.12%, which was much higher than the desired value of 80%. Thus, a total of 100 patients were selected based on the expected rate of patient visits and the inclusion criteria. The sample size calculation was adjusted for a potential 20% attrition rate to account for any dropouts or incomplete data. This sample size was deemed adequate to achieve statistically significant results while maintaining a practical patient load over the one-year study period.

Recruitment

Patients were recruited from the ENT outpatient department, where two to three new cases of COM presented daily. With twice-weekly OPD visits, this ensured a steady flow of new patients for inclusion in the study. Consecutive sampling was employed, meaning all eligible patients who met the inclusion criteria and provided informed consent were included in the study.

Data collection

Data were systematically recorded for each patient and included demographic details such as age, sex, occupation, and other relevant background information. A detailed medical history was obtained, including the duration of disease, the ear affected (right or left), previous treatment history, and any systemic comorbidities such as diabetes or hypertension. A thorough ENT examination was performed, focusing on tympanic membrane findings, including perforation size, the presence or absence of discharge, the presence of cholesteatoma, and any other pathologic changes indicative of squamosal or mucosal types of COM. Hearing impairment was assessed in detail using pure-tone audiometry (PTA), which helped categorize the extent of hearing loss and correlate it with the type and severity of COM. A complete blood count (CBC) was performed for all patients, including analysis of neutrophil, lymphocyte, and platelet counts. No blinding was applied to either of the groups, investigators, or analysts.

Group classification

The patients were classified into two groups based on their infective status and type of COM. Based on infective status, cases were defined as active when characterized by the presence of discharge and inactive when characterized by the absence of discharge for more than six months. Based on the type of COM, group 1 included patients with squamosal disease, characterized by the presence of cholesteatoma or squamous epithelial migration, whereas group 2 included patients with mucosal disease, characterized by chronic inflammation of the mucosa without cholesteatoma formation.

Blood sample collection

Presurgery Sampling

Blood samples were collected from each patient before surgery to ensure that the inflammatory markers reflected the baseline inflammatory status and were not influenced by the surgical process.

Sample Handling

Blood samples were collected in EDTA vials to prevent clotting and were processed within a few hours of collection for CBC analysis. Each vial was labeled with a unique identification number and sent to the pathology laboratory, where the samples were analyzed using one of two automated analyzers: Sysmex NX 1000 (Sysmex Corporation, Kobe, Hyōgo) or Mindray 2500 (Shenzhen Mindray Bio‑Medical Electronics Co., Ltd., Shenzhen, Guangdong) models. These analyzers process whole blood and generate results within 30 seconds of initiation. The output, decoded by computers attached to the analyzers, provides detailed counts of various blood cell types and other hematological parameters. The hospital laboratory reference ranges were used for interpretation: 1,500-7,500 cells/mm^3^ for neutrophils, 750-4,500 cells/mm^3^ for lymphocytes, and 150,000-450,000 cells/mm^3^ for platelets.

Inflammatory markers

The systemic inflammatory markers were calculated based on the CBC data. The SII was determined by multiplying the platelet and neutrophil counts and dividing the result by the lymphocyte count. The NLR was calculated by dividing the neutrophil count by the lymphocyte count. The PLR was computed by dividing the platelet count by the lymphocyte count.

Statistical analysis

Tools

Statistical analyses were carried out using Jamovi version 2.3.28 (The jamovi Project, Sydney, Australia) on the Windows 10 operating system (Microsoft Corporation, Redmond, Washington). Receiver operating characteristic (ROC) curve analysis was performed to evaluate the diagnostic performance of the biomarkers in distinguishing between patients with and without the disease. The outcome variable was binary, with disease status categorized as squamosal or mucosal. The biomarker levels, which were continuous, served as the predictor variables, and different cutoff values were tested to assess the trade-off between sensitivity and specificity. The sample size was sufficient (n = 100), and the data showed adequate variability in biomarker levels across subjects. The classifier had been validated previously in a different cohort, and the assumptions of independence and binary outcome were met in the study design.

Descriptive Statistics

The baseline demographic, clinical, and laboratory data were summarized using mean, standard deviation (SD), and other appropriate descriptive statistics.

ROC Curve Analysis

The optimal cutoff values for SII, NLR, and PLR in predicting the efficacy and prognosis of treatment were determined using ROC curve analysis. This technique facilitated the identification of threshold values that best discriminated between favorable and unfavorable outcomes for patients with COM.

Area Under the Curve (AUC)

The AUC values obtained from the ROC curve analysis were used to assess the predictive performance of SII, NLR, and PLR in relation to clinical outcomes of COM, such as hearing improvement, disease resolution, and recurrence.

Comparative Analysis

Differences between the two groups (squamosal versus mucosal disease) in terms of inflammatory marker values and clinical outcomes were compared using appropriate statistical tests, including the independent t-test, Mann-Whitney U test, or Chi-square test, depending on data distribution and type.

## Results

In our study, we analyzed the age and gender characteristics of two groups of patients: group 1 with squamosal COM and group 2 with mucosal COM. Table 3 presents the demographic characteristics of the two groups, group 1 (squamosal COM) and group 2 (mucosal COM), in terms of gender and age. In group 1, there were 21 males and 15 females, while in group 2, there were 27 males and 37 females. Although there appeared to be a higher number of females in the mucosal COM group, statistical analysis using the Chi-square test revealed that the difference in gender distribution between the two groups was not statistically significant (χ² = 2.406, p = 0.121). Regarding age, the mean age in group 1 was 32.9 ± 12.3 years, while in group 2 it was 33.3 ± 11.6 years, with an overall mean age of 33.1 ± 11.8 years across all participants. The independent samples t-test showed no statistically significant difference in age between the two groups (t = 0.170, p = 0.865). These results indicate that the two groups were comparable in terms of gender and age, and these demographic factors are unlikely to influence differences observed in other clinical or study outcomes.

**Table 3:** Gender and age characteristics of the two groups COM: chronic otitis media.

Demographic Criteria	Group 1 (Squamosal COM)	Group 2 (Mucosal COM)	Total	Test Statistic	p-value
Gender					
Male	21	27	48	2.406	0.121
Female	15	37	52		
Age (years) (mean ± SD)	32.9 ± 12.3	33.3 ± 11.6	33.1 ± 11.8	0.170	0.865

Table 4 compares inflammatory markers between group 1 (squamosal COM) and group 2 (mucosal COM). Neutrophil counts were significantly higher in group 2 (4.12 ± 1.26) compared to group 1 (3.26 ± 1.28), with p = 0.004. Although lymphocyte levels were slightly lower in group 2 (2.05 ± 0.66) than in group 1 (2.31 ± 0.78), this difference was not statistically significant (p = 0.096). Platelet counts showed a highly significant increase in group 2 (313.84 ± 78.75) compared to group 1 (247.50 ± 53.08), p < 0.001. Similarly, SII was markedly elevated in group 2 (679.11 ± 301.25) compared to group 1 (362.30 ± 206.37 × 10³/cu.mm), with Welch’s t-test confirming a highly significant difference (p < 0.001). Both NLR and PLR were also significantly higher in mucosal COM (NLR: 2.14 ± 0.79, PLR: 166.16 ± 64.93) than in squamosal COM (NLR: 1.55 ± 1.20, PLR: 117.39 ± 39.47), with p = 0.001 and p < 0.001, respectively. These findings suggest a greater systemic inflammatory response in mucosal COM.

**Table 4:** : Comparison of NLR, PLR, and SII values of the two groups * Welch’s t-test; for the remaining, Student’s t-test was used. COM: chronic otitis media, SD: standard deviation, SII: systemic immune-inflammation index, NLR: neutrophil-to-lymphocyte ratio, PLR: platelet-to-lymphocyte ratio.

Parameter	Group 1 (Squamosal COM)	Group 2 (Mucosal COM)	t-test Statistic	p-value
Neutrophils (×10³/mm^3^) (mean ± SD)	3.26 ± 1.28	4.12 ± 1.26	2.950	0.004
Lymphocytes (×10³/mm^3^) (mean ± SD)	2.31 ± 0.78	2.05 ± 0.66	-1.683	0.096
Platelets (×10³/mm^3^) (mean ± SD)	247.50 ± 53.08	313.84 ± 78.75	4.507	<0.001
SII (×10³/mm^3^) (mean ± SD)	362.30 ± 206.37	679.11 ± 301.25	5.628*	<0.001
NLR (mean ± SD)	1.55 ± 1.20	2.14 ± 0.79	3.334	0.001
PLR (mean ± SD)	117.39 ± 39.47	166.16 ± 64.93	4.096	<0.001

Table 5 presents the diagnostic performance of three inflammatory markers (SII, NLR, and PLR) in differentiating types of COM. The cutoff values were determined using Youden’s Index (J), a statistical measure that helps identify the optimal cutoff point for a diagnostic test by balancing its ability to correctly identify true positives and true negatives. By maximizing J (J = sensitivity + specificity − 1), it identifies the threshold that provides the best overall diagnostic accuracy.

**Table 5:** Diagnostic performance of inflammatory markers in differentiating COM types AUC: area under the curve, CI: confidence interval, SII: systemic immune-inflammation index, NLR: neutrophil-to-lymphocyte ratio, PLR: platelet-to-lymphocyte ratio.

Group	AUC (95% CI)	Cutoff Value	p-value	Sensitivity	Specificity
SII	0.863 (0.786-0.940)	429.31	< 0.001	84.38%	75.00%
NLR	0.778 (0.682-0.874)	1.59	< 0.001	78.12%	75.00%
PLR	0.756 (0.658-0.853)	162.55	< 0.001	53.12%	86.11%

Among these markers, SII demonstrated the highest diagnostic accuracy, with an AUC of 0.863 (95% CI, 0.786-0.940), indicating excellent discriminative ability. At a cutoff value of 429.31, SII showed a sensitivity of 84.38% and specificity of 75.00% (p < 0.001), indicating that values higher than 429.31 are suggestive of mucosal COM.

NLR also showed good diagnostic potential, with an AUC of 0.778 (95% CI, 0.682-0.874), sensitivity of 78.12%, and specificity of 75.00% at a cutoff of 1.59 (p < 0.001), suggesting that values below 1.59 point to squamosal COM.

**Figure 1:**
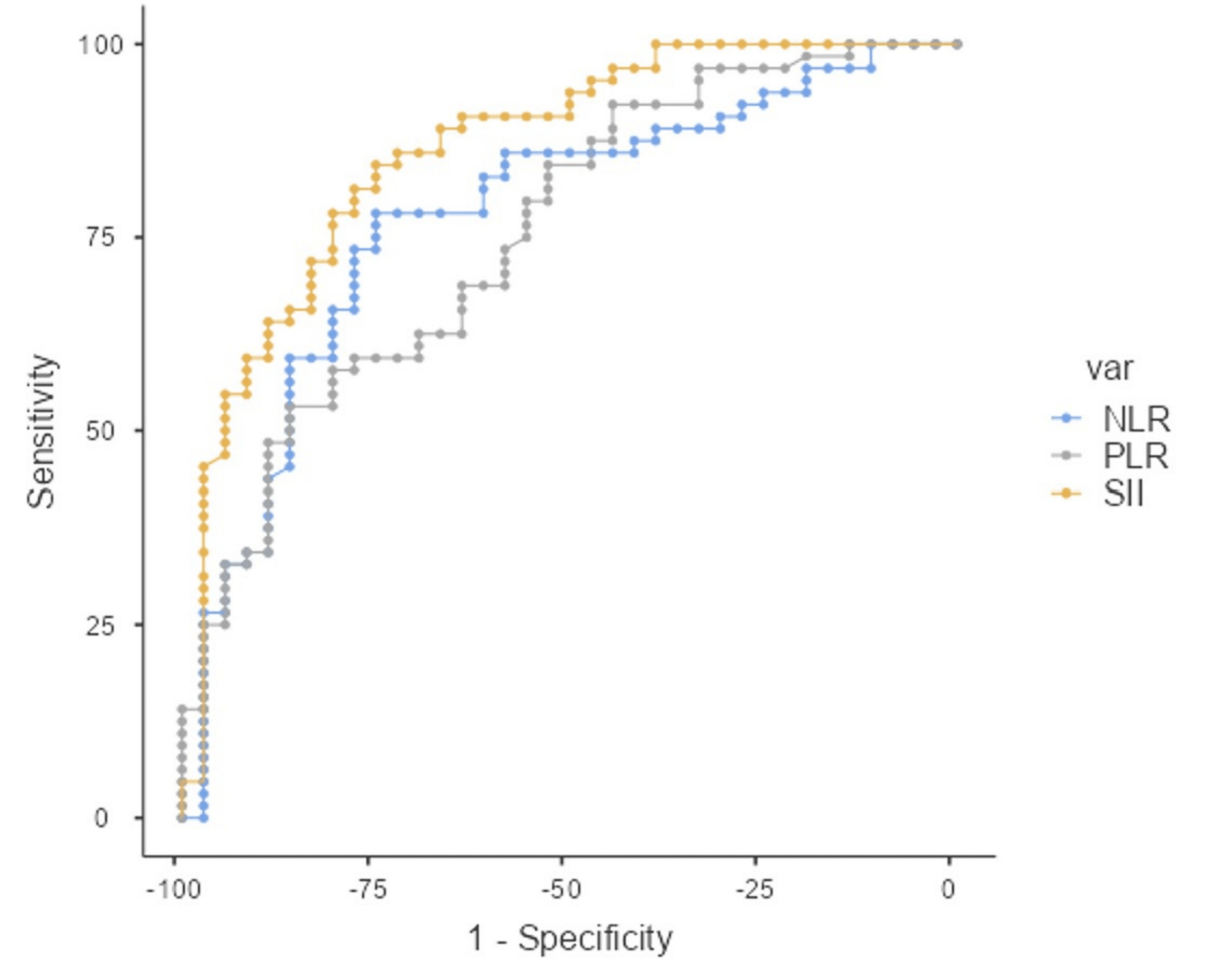
Combined ROC curves for NLR, PLR, and SII in differentiating squamosal and mucosal chronic otitis media The figure illustrates the ROC Curves for SII, NLR, and PLR with AUCs of 0.863, 0.778, and 0.756, respectively, at cutoff values of 429.31, 1.59, and 162.55, respectively. SII, NLR, and PLR values higher than the cutoff values suggest mucosal COM and squamosal COM when they are below the cutoff values. ROC: receiver operating characteristic, NLR: neutrophil-to-lymphocyte ratio, PLR: platelet-to-lymphocyte ratio, SII: systemic immune-inflammation index, AUC: area under the curve, COM: chronic otitis media.

PLR had the lowest diagnostic value, with an AUC of 0.756 (95% CI, 0.658-0.853). Although it achieved high specificity (86.11%) at a cutoff of 162.55, its sensitivity was considerably lower at 53.12% (p < 0.001), with values higher than 162.55 indicating a potential diagnosis of mucosal COM. Overall, SII appears to be the most effective marker for distinguishing COM types, followed by NLR and PLR (Figure 1).

## Discussion

COM is a persistent inflammatory condition of the middle ear, commonly categorized into squamosal and mucosal subtypes based on its underlying pathophysiology and clinical behavior. While squamosal COM is typically associated with local destructive processes such as cholesteatoma formation, mucosal COM often reflects ongoing systemic inflammation [27,28]. In our study, we aimed to evaluate and compare the systemic inflammatory status of patients with squamosal and mucosal COM using three widely available and cost-effective hematological indices: NLR, PLR, and SII.

Our results demonstrated that patients with mucosal COM exhibited significantly higher values of NLR, PLR, and SII compared to those with squamosal COM. These findings suggest that mucosal COM is associated with a more pronounced systemic inflammatory response. In contrast, squamosal COM showed relatively lower systemic inflammatory indices, supporting the notion that its inflammatory impact is more localized.

Several previous studies have highlighted the diagnostic and prognostic utility of NLR in various inflammatory and infectious conditions, including otologic diseases such as otitis media with effusion, Bell’s palsy, and sensorineural hearing loss [29-35]. Our findings align with these observations and extend them by showing that NLR values were significantly elevated in mucosal COM compared to squamosal COM. However, while earlier studies suggested that NLR lacks predictive value in assessing the severity or complications of cholesteatoma [36-38], our results emphasize its relevance in differentiating between COM subtypes.

PLR, although studied less extensively in otology, has also been proposed as a marker of systemic inflammation [39]. In our cohort, PLR was significantly higher in the mucosal group, with a specificity of over 90% in differentiating mucosal from squamosal COM. This reinforces its potential clinical value, particularly when used alongside other markers.

SII, a newer index combining platelet, neutrophil, and lymphocyte counts, demonstrated the highest diagnostic performance among the three indices in our study. With an AUC of 0.894 and balanced sensitivity and specificity, SII appears to be a robust and practical tool for COM subtype differentiation, as showcased in other studies as well [39]. Unlike cytokine panels or other advanced inflammatory assays, SII is inexpensive, reproducible, and can be readily calculated from a routine complete blood count. Despite its extensive use in oncology and systemic inflammatory diseases, literature on its application in otologic conditions is scarce. Our study is among the first to highlight its potential role in otitis media classification.

These findings support the idea that mucosal COM may reflect systemic inflammation, whereas squamosal COM remains more localized in nature. Thus, inflammatory indices, particularly SII, could serve as important adjunctive tools in preoperative assessment and management decisions.

However, our study is not without limitations. Being a single-center study with a relatively small sample size limits the generalizability of our findings. Moreover, disease activity (active versus inactive) was not uniformly classified across all cases, which may have introduced variability in inflammatory marker levels.

Study limitations

This study has several limitations that may affect the validity and generalizability of its findings. Conducting the research in a single tertiary care hospital restricts its applicability, as patient demographics, clinical practices, and resource availability may differ across other settings, particularly in lower-resource environments. Additionally, although the study accounted for a 20% attrition rate, loss to follow-up and noncompliance could introduce bias, especially if those who dropped out differed meaningfully from those who remained. The observational, nonrandomized design also increases susceptibility to selection bias related to variability in infective status of the disease and confounding, as unmeasured variables and baseline differences may influence both inflammatory marker levels and clinical outcomes, limiting causal interpretations. Furthermore, inconsistencies in how inflammatory markers were measured or recorded, along with reliance on infrequent or single-point testing, may have resulted in measurement bias and may not accurately reflect the dynamic nature of inflammatory processes.

## Conclusions

In conclusion, our study suggests that systemic inflammatory markers, especially the SII, hold significant potential in differentiating between mucosal and squamosal types of COM. SII, being a simple, cost-effective, and easily obtainable parameter, demonstrated superior diagnostic performance compared to NLR and PLR. These results support the hypothesis that mucosal COM is associated with a stronger systemic inflammatory response, whereas squamosal COM is likely characterized by localized inflammation. Incorporating SII into routine preoperative evaluation may aid in the early differentiation of COM subtypes and guide clinical decision-making.

Further large-scale, prospective studies are warranted to validate these findings and to establish standard reference values for SII and other inflammatory indices in the context of otologic diseases.
